# Glycogen Synthase Kinase 3 Beta Regulates the Human Aryl Hydrocarbon Receptor Cellular Content and Activity

**DOI:** 10.3390/ijms22116097

**Published:** 2021-06-05

**Authors:** Yujie Yang, William K. Chan

**Affiliations:** Department of Pharmaceutics & Medicinal Chemistry, Thomas J. Long School of Pharmacy, University of the Pacific, Stockton, CA 95211, USA; y_yang16@u.pacific.edu

**Keywords:** aryl hydrocarbon receptor, GSK3β, LC3, autophagy, lysosomal degradation, p23

## Abstract

The aryl hydrocarbon receptor (AHR) is a cytosolic receptor which is involved in diverse cellular events in humans. The most well-characterized function of AHR is its ability to upregulate gene transcription after exposure to its ligands, such as environmental toxicants, dietary antioxidants, drugs, and endogenous ligands. The cellular content of AHR is partly controlled by its degradation via the ubiquitin–proteasome system and the lysosome-dependent autophagy. We used human cervical cancer (HeLa) cells to investigate how AHR undergoes protein degradation and how its activity is modulated. Since the glycogen synthase kinase 3 beta (GSK3β)-mediated phosphorylation can trigger protein degradation and substrates of GSK3β contain stretches of serine/threonine residues which can be found in AHR, we examined whether degradation and activity of AHR can be controlled by GSK3β. We observed that AHR undergoes the GSK3β-dependent, LC3-mediated lysosomal degradation without ligand treatment. The AHR can be phosphorylated in a GSK3β-dependent manner at three putative sites (S436/S440/S444, S689/S693/T697, and S723/S727/T731), which leads to lysosomal degradation of the AHR protein. Inhibition of the GSK3β activity suppresses the ligand-activated transcription of an AHR target gene in HeLa, human liver cancer (Hep3B), and human breast cancer (MCF-7) cells. Collectively, our findings support that phosphorylation of AHR by GSK3β is essential for the optimal activation of its target gene transcription and this phosphorylation may partake as an “off” switch by subjecting the receptor to lysosomal degradation.

## 1. Introduction

The aryl hydrocarbon receptor (AHR) is a cellular sensor of environmental pollutants/carcinogens, endogenous ligands, and numerous dietary flavonoids and their derivatives [1]. Upon ligand binding, the AHR cytosolic complex changes conformation to reveal its nuclear localization motif, resulting in nuclear entry. The nuclear AHR dimerizes with aryl hydrocarbon receptor nuclear translocator (ARNT) to form an active transcription factor: it binds to the dioxin response element, recruits co-activators locally to alter chromatin structure, and eventually allows optimal assembly of the preinitiation complex for gene transcription. Although AHR is best known for its action as a ligand-activated transcription factor which regulates transcription of xenobiotic metabolizing enzyme genes, the biological role of this receptor is rather complex, and yet very interesting. For example, recent reports show that AHR plays an intricate role in seemingly diverse biological processes and diseases, namely autoimmune diseases [2,3,4], cancers and cell proliferation [5,6,7,8,9], cancer stem cell differentiation [10], respiratory disorders [11,12], atopic dermatitis [13,14,15], bone disorders [16], antiviral response [17], and adipocyte differentiation [18]. This receptor is even implicated in the SARS-CoV-2 pathophysiology [19]. Obviously AHR has become an attractive target for drug development; thus, any means to modulate its function in selective tissues is highly desirable. AHR normally translocates into the nucleus after ligand binding, followed by degradation via the ubiquitin-proteasome system [20,21,22]. Interestingly, even without addition of an exogenous ligand, we showed that AHR undergoes lysosomal degradation in various untransformed and immortalized cell lines [23,24]. An in-depth understanding of the regulation of the cellular AHR content should unveil ways to modulate the AHR function by controlling its protein levels.

Glycogen synthase kinase 3 beta (GSK3β) is a serine/threonine kinase which was first discovered to regulate gluconeogenesis by phosphorylation of glycogen synthase [25]. Since then, more than five hundred GSK3β substrates have been suggested based on the motif-based predictions [26], which make this kinase one of the most complicated kinases for cellular processes. One of its most notable substrates is β-catenin. Upon phosphorylation by GSK3β, β-catenin is degraded via the ubiquitin-proteasome system; GSK3β is therefore recognized as a key player in the regulation of β-catenin function, particularly in the promotion of cancer growth [27]. Our laboratory is interested in studying the underpinnings of AHR degradation in controlling its basal levels within a cell. Realizing that GSK3β is responsible for β-catenin degradation, we hypothesized that events such as GSK3β phosphorylation may play a role in AHR degradation since the carboxyl region of AHR is serine and threonine rich. Here, we provide evidence supporting that GSK3β is responsible for AHR phosphorylation, which directs AHR to lysosomal degradation. This phosphorylation is essential for the optimal transcription of AHR target genes upon ligand activation.

## 2. Results

### 2.1. GSK3β Regulates the AHR Protein Levels in HeLa Cells

**Figure 1:**
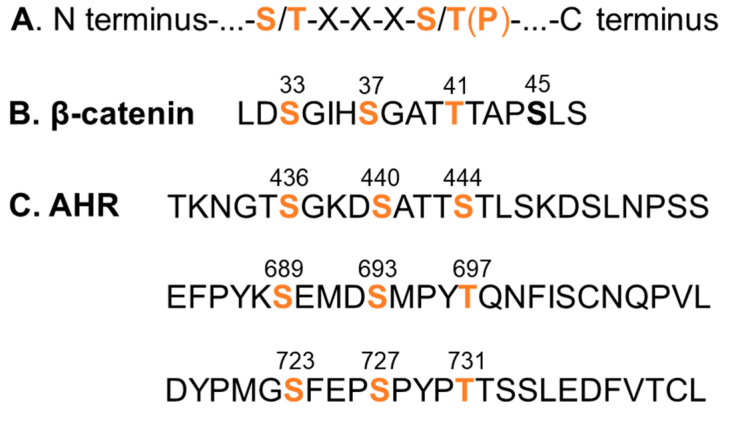
GSK3β phosphorylation sites. (**A**) GSK3β phosphorylation consensus sequence. (**B**) GSK3β phosphorylation sites of β-catenin. (**C**) Three putative GSK3β phosphorylation sites of the human AHR (amino acid 1-848) which show best match to the consensus sequence. The numbers indicate the amino acid locations of the primary sequence. Orange font represents the serine and threonine sites for GSK3β phosphorylation. S45 (bold) of β-catenin represents the priming site of casein kinase 1. Phosphorylation of the priming site is necessary for GSK phosphorylation.

**Figure 2:**
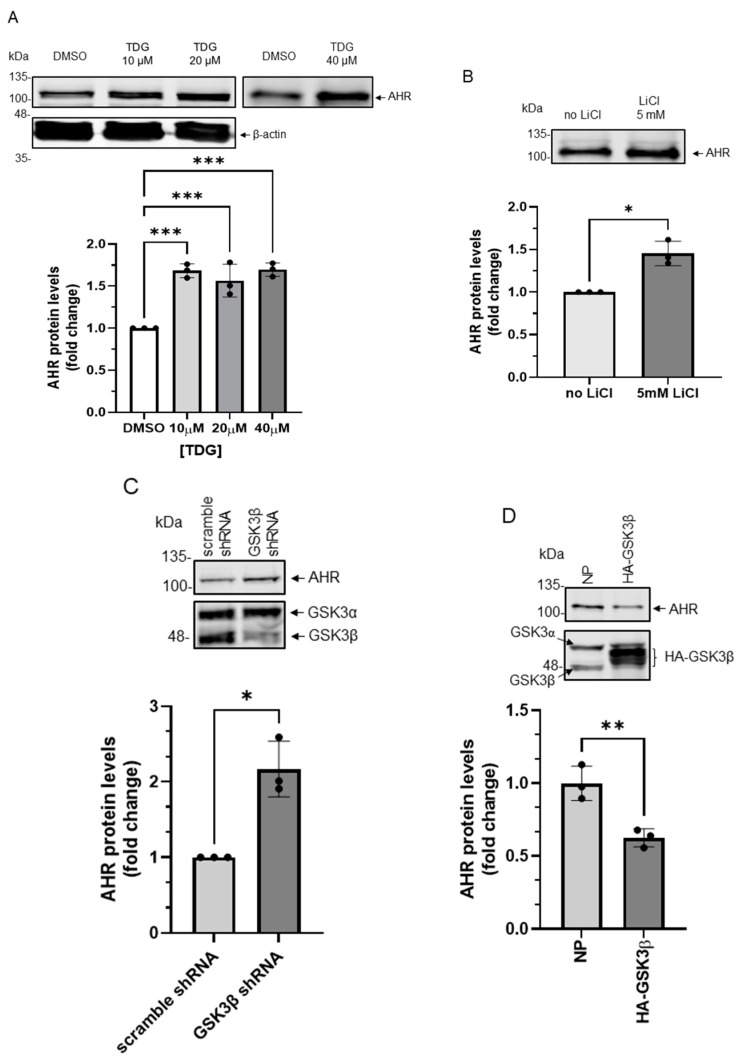
AHR protein levels are regulated by GSK3β in HeLa cells. (**A**) HeLa cells were treated with 10 μM, 20 μM or 40 μM TDG for 8 h. Western blot analysis showed that AHR protein levels increased after TDG treatment. The images are representative of the replicate data (means ± SD, *n* = 3, *** *p* < 0.001). DMSO group (treated for 8 h) was arbitrarily set as one (with no error bar) for data normalization. One-way ANOVA with Dunnett multiple comparisons test was performed to determine statistical significance. (**B**) AHR protein levels increased after 5 mM LiCl treatment for 6 h. The images are representative of the replicate data (means ± SD, *n* = 3, * *p* < 0.05). No LiCl (no treatment) group was arbitrarily set as one (with no error bar) for data normalization. Unpaired *t*-test with Welch’s correction was used to determine the statistical significance. (**C**) Transient knockdown of GSK3β (after 72 h) increased AHR protein levels. The images are representative of the replicate data (means ± SD, *n* = 3, * *p* < 0.05). Cells transfected with scramble shRNA were used as the negative control and was arbitrarily set as one (with no error bar) for data normalization. Unpaired *t*-test with Welch’s correction was used to determine the statistical significance. (**D**) Transient expression of HA-GSK3β (after 72 h) decreased AHR protein levels. The images are representative of the replicate data (means ± SD, *n* = 3, ** *p* < 0.01) in one experiment and was repeated two more times with similar results. No plasmid group (NP) represents cells undergoing the same transient transfection without a plasmid. Unpaired *t*-test was used to determine the statistical significance. For A to D, each Western lane contained 30 μg of whole-cell lysate. Data in A for 10-20 μM treatment groups were normalized by β-actin (as shown) whereas the rest of the data were normalized by total protein stain.

When we examined the GSK3β phosphorylation sites of β-catenin and the general requirement of a GSK3β substrate [28], we identified three similar stretches of serine/threonine regions of the human AHR which would hint AHR as a potential substrate of GSK3β (Figure 1). With the understanding that the phosphorylated β-catenin by GSK3β undergoes protein degradation, we first examined whether the AHR protein levels can be reduced by GSK3β. After treating HeLa cells with 10-40 μM tideglusib (TDG), which is a specific GSK3β inhibitor [29], AHR protein levels were significantly increased to 1.7-fold (Figure 2A). Another GSK3β inhibitor lithium chloride (LiCl) also significantly increased the AHR protein levels to 1.5-fold (Figure 2B). Downregulation of GSK3β by a sequence-specific shRNA significantly increased the AHR protein levels to two-fold when compared to the scramble shRNA negative control (Figure 2C). The AHR protein levels were significantly suppressed to 60% of the wild-type content when the HA-tagged GSK3β was transiently expressed (Figure 2D). Collectively, these results strongly supported that GSK3β downregulates the AHR protein content in HeLa cells.

### 2.2. GSK3β Causes the Autophagy-Mediated Lysosomal Degradation of AHR in HeLa Cells

**Figure 3:**
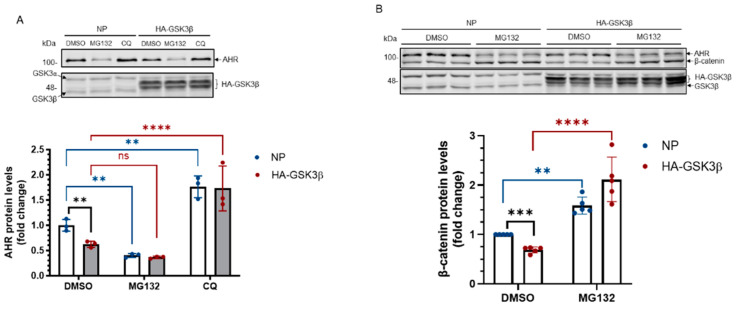

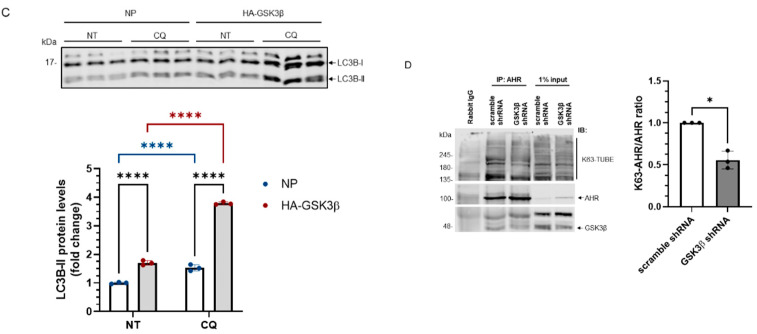
AHR undergoes the LC-3-mediated autophagy after GSK3β phosphorylation. (**A**) AHR protein levels ± HA-GSK3β transient expression (72 h) were increased by CQ (40 μM for 6 h, last 6 h of the 72-h period of transient transfection) but not by MG132 (10 μM for 6 h, last 6 h of the 72-h period of transient transfection). The time and dose of CQ and MG132 were optimized for inhibition of degradation. The images are representative of the replicate data (means ± SD, *n* = 3, ** *p* < 0.01, **** *p* < 0.0001, ns represents not significant) in one experiment and was repeated once with similar results. DMSO treatment group (treated for 6 h) was arbitrarily set as one for data normalization. No plasmid (NP) group represents cells undergoing the same transient transfection as HA-GSK3β but without plasmid. Two-way ANOVA with Dunnett multiple comparisons test was performed to determine statistical significance. (**B**) β-catenin protein levels decreased after HA-GSK3β transient expression (72 h) and were increased ± HA-GSK3β by MG132 (10 μM for 6 h, last 6 h of the 72-h period of transient transfection). The images are representative of the replicate data (means ± SD, *n* = 5, ** *p* < 0.01, *** *p* < 0.001, **** *p* < 0.0001). DMSO treatment group was arbitrarily set as one for data normalization. No plasmid (NP) group represents cells undergoing the same transient transfection as HA-GSK3β but without plasmid. Two-way ANOVA with Sidak multiple comparisons test was performed to determine statistical significance. (**C**) LC3B-II levels with HA-GSK3β transient expression (72 h) were higher than that in the no plasmid group (NP). No plasmid (NP) group represents cells undergoing the same transient transfection as HA-GSK3β but without plasmid. The fold change of LC3B-II increase after 6-h, 40 μM CQ treatment was also higher in the HA-GSK3β group. The images are representative of the replicate data (means ± SD, *n* = 3, **** *p* < 0.0001) in one experiment and was repeated once with similar results. No treatment (NT) group (treated for 6 h) was arbitrarily set as one for data normalization. Two-way ANOVA with Tukey multiple comparisons test was performed to determine statistical significance. (**D**) Immunoprecipitation of AHR from HeLa whole cell lysates ± GSK3β knockdown using anti-AHR SA210 polyclonal antibody, followed by K63-TUBE Far-western analysis showing less K63-ubiquitinated AHR in GSK3β knockdown group while more AHR (104 kDa) being precipitated when compared to the scramble shRNA-transfected group. The images are representative of the replicate data (means ± SD, *n* = 3, * *p* < 0.05) with scramble shRNA group set as one for normalization of three separate experiment (thus no error bar). For A to C, each Western lane contained 30 μg of whole-cell lysate. Data were normalized by total protein stain.

Next, we examined whether GSK3β would suppress the AHR levels by promoting AHR degradation. We performed experiments using a proteasome inhibitor MG132 and an autophagy inhibitor chloroquine (CQ). We observed that MG132 surprisingly decreased the amount of AHR protein in HeLa cells that were transiently transfected with either a plasmid carrying the HA fusion of GSK3β cDNA or with no plasmid (Figure 3A). CQ, however, increased the AHR protein levels in HeLa cells with or without HA-GSK3β expression (Figure 3A), suggesting that AHR undergoes the GSK3β-dependent lysosomal degradation, but not proteasomal degradation, in HeLa cells without addition of an AHR ligand. Treatment of CQ increased the AHR protein levels in the HeLa cells which had undergone transfection but with no plasmid, suggesting that AHR is actively undergoing lysosomal degradation. To ensure that on our hand, MG132 could inhibit proteasomal degradation in Figure 3A, we examined the change of the β-catenin protein levels in HeLa cells and confirmed that MG132 reversed the GSK3β-mediated reduction of the β-catenin levels (Figure 3B). Next, we examined whether GSK3β could trigger autophagy in HeLa cells by measuring the LCB-II levels, which correspond to the autophagic flux. We observed that the LC3B-II protein levels in HA-GSK3β expressing cells were increased (1.7-fold) when compared to cells that had undergone transfection with no plasmid (Figure 3C, NT). After 6 h of CQ treatment, the increase of LC3B-II was much higher in the HA-GSK3β group, suggesting that GSK3β causes an increase of the autophagic flux in HeLa cells. Next, we examined whether the K63-ubiquitination of AHR would occur, which might lead to its lysosomal degradation. We performed Far-western analysis using lysine (K) 63 specific tandem ubiquitin binding entity (TUBE) conjugated to biotin. This K63 TUBE reagent has a 1000-10,000-fold binding affinity favoring the K63-linked ubiquitin than the other linkages. After immunoprecipitation using the SA-210 AHR antibody, GSK3β knockdown lysate showed higher amount of AHR while the K63-ubiquitinated AHR was less when compared to the scramble shRNA transfected group (Figure 3D). The ratio of K63-ubiquitinated AHR/total AHR decreased after GSK3β knockdown, supporting that GSK3β promotes the K63-ubiquitination of AHR. Collectively, our results support that GSK3β causes the AHR degradation in lysosome via the K63-dependent, LC3-mediated autophagy.

### 2.3. GSK3β Phosphorylates AHR in HeLa Cells

**Figure 4:**
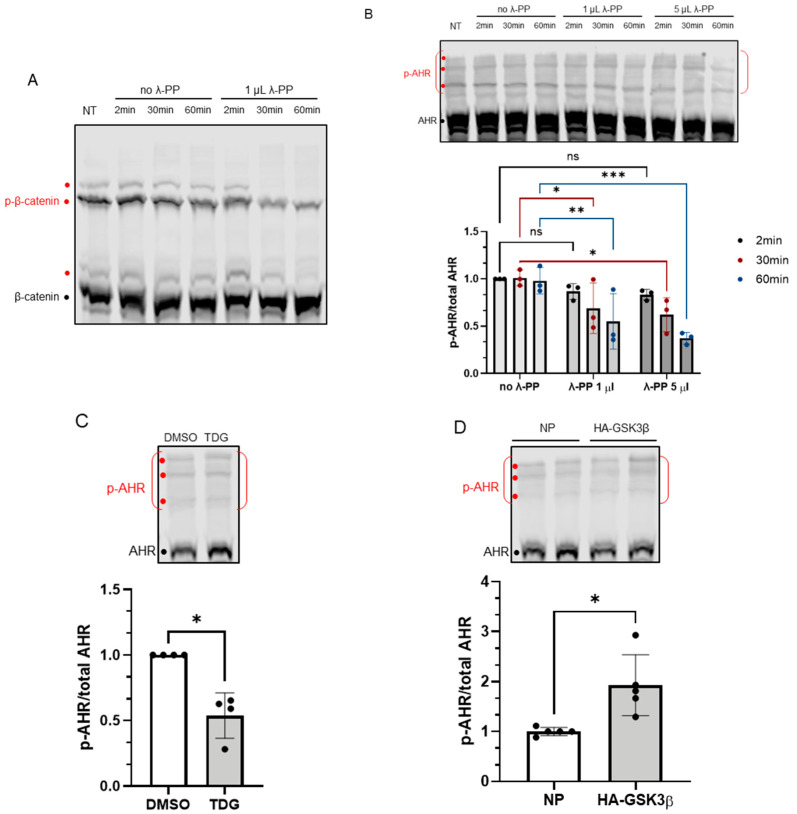
Phosphorylation of AHR is affected by GSK3β activity. 7.5% acrylamide bis-Tris gel embedded with 20 μM phos-tag reagent was used to confirm the phosphorylation of AHR. (**A**) Phosphorylated (p-β-catenin) and unphosphorylated β-catenin can be separated, and the intensities of three phosphorylated bands (red dots) were noticeably decreased by 1 μL (80 units) of λ-PP treatment for 2, 30 or 60 min at 30 °C. NT represents no treatment. The top region of AHR blot in B-D shows three prominent phosphorylated AHR bands (p-AHR, red dots). The whole top region indicated by the red bracket is defined as the phosphorylated region and unphosphorylated AHR is indicated with the black dot. The y-axis (p-AHR/total AHR) represents intensity of the phosphorylated bracket region divided by the sum of the intensities of the bracket region plus unphosphorylated AHR. (**B**) Time- and dose-dependent decrease of the p-AHR/total AHR value after 1 μL (80 units) or 5 μL (400 units) of λ-PP treatment for 2, 30 or 60 min for 30 °C. The images are representative of the replicate data (means ± SD, *n* = 3, * *p* < 0.05, ** *p* < 0.01, *** *p* < 0.001, ns represents not significant). Two-minute of no λ-PP treatment group was arbitrarily set as one (with no error bar) for data normalization. Two-way ANOVA with Dunnett multiple comparisons test was performed to determine statistical significance. (**C**) After 40 μM TDG treatment for 8 h, the p-AHR/total AHR values decreased. The images are representative of the replicate data (means ± SD, *n* = 4, * *p* < 0.05). DMSO group (treated for 8 h) was arbitrarily set as one (with no error bar) for data normalization. Unpaired *t*-test with Welch’s correction was used to determine the statistical significance. (**D**) Transient expression of HA-GSK3β increased the p-AHR/total AHR value. The images are representative of the replicate data (means ± SD, *n* = 5, * *p* < 0.05). No plasmid group (NP), which represents cells undergone same transient transfection protocol without the plasmid carrying HA-GSK3β cDNA, was arbitrarily set as one for data normalization. Unpaired *t*-test with Welch’s correction was used to determine the statistical significance. For A to D, each Western lane contained 40 μg of whole-cell lysate. Data were normalized by total protein stain.

Next, we examined whether AHR could be phosphorylated by GSK3β. Phos-tag reagent, which has been successfully used by other researchers to detect phosphorylated proteins [30,31], was used to detect the phosphorylated AHR. In principle, phos-tag reagent binds selectively to the phosphate group(s) of protein and retards the bound protein during electrophoresis when the reagent was embedded evenly in the acrylamide gel. First, we confirmed that the phosphorylated β-catenin could be detected using this approach with a 7.5% acrylamide gel containing 20 μM phos-tag reagent (Figure 4A). Then we used the same strategy to detect the phosphorylated AHR. There were three bands detected using the anti-AHR SA-210 antibody at the top region of the immunoblot, indicated by red dots (Figure 4B). These higher mobility bands were not observed in a conventional acrylamide gel without phos-tag reagent. Intensity of the putative phosphorylated AHR region showed a time- and dose-dependent decrease after phosphatase (λ-PP) treatment, supporting that these bands represent the phosphorylated AHR (Figure 4B). After λ-PP treatment, the top region intensities were weaker while the unphosphorylated AHR band intensity increased. Thus, we quantified our data by the ratio of the intensity of the phosphorylated region to the intensity of the total AHR, which was the combined intensity of phosphorylated and unphosphorylated AHR. Inhibition of GSK3β by TDG yielded more unphosphorylated AHR and showed that the phosphorylated AHR/total AHR value decreased to about 50% (Figure 4C). Transient expression of the HA fusion of GSK3β increased the phosphorylated AHR region intensity and decreased the unphosphorylated AHR intensity. The phosphorylated AHR/total AHR value increased to 2-fold when HA-GSK3β was transiently expressed (Figure 4D). Taken together, AHR can be phosphorylated in a GSK3β-dependent manner.

### 2.4. Mutational Analysis Reveals Three Putative GSK3β Phosphorylation Regions of AHR

**Figure 5:**
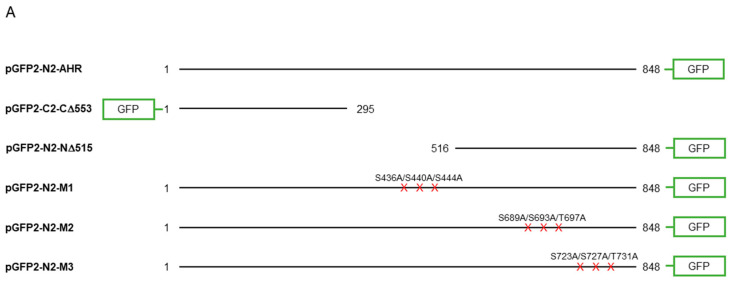

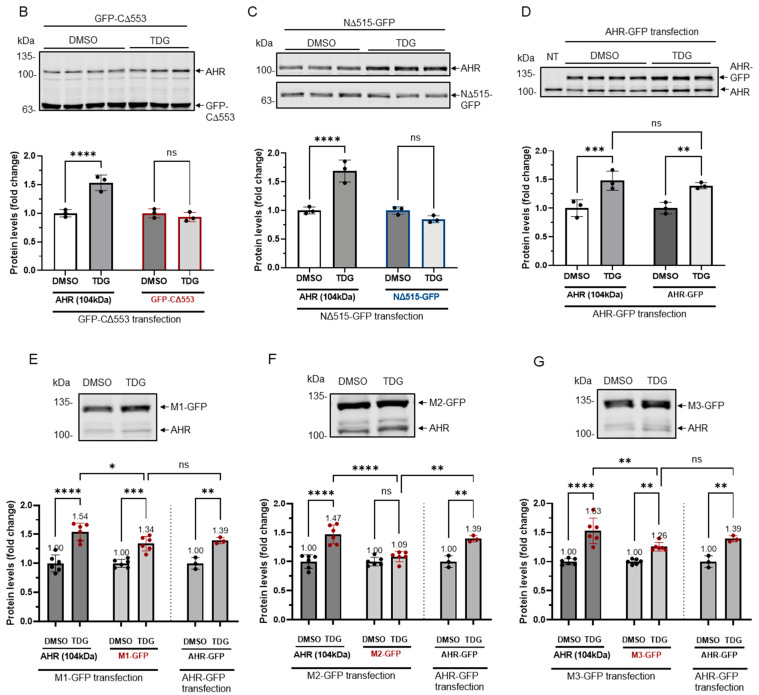
Site-directed mutagenesis reveals three putative GSK3β phosphorylation sites of AHR. (**A**) A map showing GFP fusion expressing plasmids of full length human AHR and two truncated derivatives (pGFP2-N2-AHR, pGFP2-C2-CΔ553, and pGFP2-N2-NΔ515) and three AHR mutant GFP plasmids (S436A-S440A-S444A, M1; S689A-S693A-T697A, M2; S723A-S727A-T731A, M3). The names of the plasmid expressing the corresponding protein are listed on the left. Both GFP fusions of CΔ553 (**B**) and NΔ515 (**C**), 56 h after transient transfection, did not respond to TDG while the full-length AHR-GFP after transiently transfecting pGFP2-N2-AHR plasmid for 56 h (**D**) showed similar increase as the endogenous AHR proteins after TDG (40 μM, 8 h) treatment. The images are representative of the replicate data (means ± SD, *n* = 3, ** *p* < 0.01, *** *p* < 0.001, **** *p* < 0.0001, ns represents not significant) in one experiment, which was repeated once with similar results. DMSO treatment group was arbitrarily set as one for data normalization. NT represents no transfection. One-way ANOVA with Sidak multiple comparisons test was performed to determine statistical significance. (**E**) M1-GFP mutant showed smaller increase (by 20%) when compared to endogenous AHR but similar increase as AHR-GFP (data from 5D) after TDG (40 μM, 8 h) treatment. (**F**) For M2-GFP mutant, its increase was minimal after treatment of 40 μM TDG for 8 h. The increased level was noticeably less when compared to AHR-GFP (data from 5D). (**G**) M3-GFP mutant showed 27% less increase when compared to the endogenous AHR and 13% less to AHR-GFP (data from 5D) after TDG (40 μM, 8 h) treatment. The images in (E-G) are representative of the replicate data (means ± SD, *n* = 6, * *p* < 0.05, ** *p* < 0.01, *** *p* < 0.001, **** *p* < 0.0001, ns represents not significant). DMSO treatment groups were arbitrarily set as one for data normalization. Two experiments of *n* = 3 each were performed so that error bars are present on the DMSO groups. The dotted lines separate the AHR-GFP transfection data that are the same data set as in 5D for easy reference. One-way ANOVA with Sidak multiple comparisons test was performed to determine statistical significance. For B to G, each Western lane contained 30 μg of whole-cell lysate. Data were normalized by total protein stain. NΔ515-GFP (5C) was detected by the GFP antibody since this construct cannot be detected by SA210 AHR antibody while all other GFP fusions of AHR and derivatives were detected by SA210 AHR antibody.

To further investigate whether AHR would be a substrate of GSK3β, we used two truncated human AHR, namely CΔ553 (amino acid 1–295) and NΔ515 (amino acid 516-848), that were fused to GFP to approximate the phosphorylation location(s) (Figure 5A). Neither of these two GFP fusions responded to TDG inhibition (Figure 5B,C) whereas both the endogenous AHR and GFP-AHR controls did (Figure 5B–D). There are three stretches of serine and threonine residues of AHR that fulfill the requirement of an GSK3β substrate: they are S436/S440/S444, S689/S693/T697, and S723/S727/T731 (Figure 1). These locations match the consensus GSK3β recognition motif and the GSK3β phosphorylation sites of β-catenin. We mutated these serine and threonine residues to alanine and generated three GFP fusion mutants, namely M1 (S436A/S440A/S444A), M2 (S689A/S693A/T697A), and M3 (S723A/S727A/T731A) (Figure 5A). After treatment of 40 μM TDG, only HeLa cells transfected with the M2-GFP fusion showed no statistically significant increase whereas M1- and M3-GFP fusions showed modest increase after GSK3β inhibition by TDG (1.26- to 1.34-fold), when compared to the endogenous AHR and AHR-GFP fusion with a somewhat higher increase upon TDG treatment (1.39- to 1.54-fold) (Figure 5E–G).

**Figure 6:**
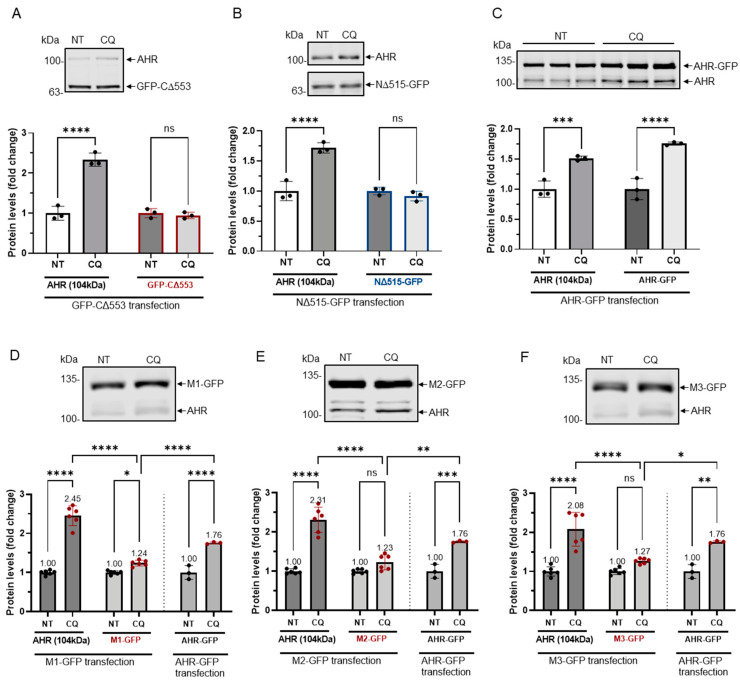
GFP fusions of two truncated AHR and three mutants were less sensitive to lysosomal degradation. HeLa cells that were transiently transfected (for 54 h) with the plasmid expressing GFP-CΔ553 (**A**), NΔ515-GFP (**B**) or full length AHR-GFP (**C**) were treated with either no treatment (NT) or 40 μM CQ for 6 h. Only AHR-GFP showed similar increase as the endogenous AHR upon TDG treatment. The images are representative of the replicate data (means ± SD, *n* = 3, *** *p* < 0.001, **** *p* < 0.0001, ns represents not significant) in one experiment, and was repeated once with similar results. No treatment group (NT) was arbitrarily set as one for data normalization. One-way ANOVA with Sidak multiple comparisons test was performed to determine statistical significance. HeLa cells that were transiently transfected (for 54 h) with the plasmid expressing M1-GFP (**D**), M2-GFP (**E**) or M3-GFP (**F**) showed a significant lower increase of 1.2- to 1.3-fold when compared to 2.0- to 2.5-fold increase of endogenous AHR upon treatment of 40 μM CQ for 6 h. These increases were significantly less when compared to AHR-GFP (data from 6C). The images in (D-F) are representative of the replicate data (means ± SD, *n* = 6, * *p* < 0.05, ** *p* < 0.01, *** *p* < 0.001, **** *p* < 0.0001). No treatment groups (NT) were arbitrarily set as one for data normalization. Two experiments of *n* = 3 each were performed so that error bars are present on the NT groups. The dotted lines separate the AHR-GFP transfection data that are presented in 6C for easy reference. One-way ANOVA with Sidak multiple comparisons test was performed to determine statistical significance. For A to F, each Western lane contained 30 μg of whole-cell lysate. Data were normalized by total protein stain. NΔ515-GFP (6B) was detected by the GFP antibody since this construct could not be detected by SA210 AHR antibody while all other GFP fusions of AHR and derivatives were detected by SA210 AHR antibody.

Next, we examined whether resistance to GSK3β control would translate into resistance to lysosomal degradation. Our results showed that levels of GFP fusions of CΔ553 and NΔ515 after transient transfection into HeLa cells were not altered after CQ, which is an autophagy inhibitor, treatment whereas the endogenous AHR and AHR-GFP fusion showed higher levels after CQ treatment (Figure 6A–C). In addition, unlike the endogenous AHR in HeLa cells which showed about 2–2.5-fold increase upon CQ treatment, levels of the GFP fusions of all three mutants (M1-M3) after transient transfection were only modestly increased (1.23- to 1.27-fold) (Figure 6D–F) while the AHR-GFP levels were increased by 1.8-fold in the presence of CQ. Collectively, these results revealed three putative GSK3β phosphorylation regions of AHR which are essential for the lysosomal degradation of AHR.

### 2.5. p23 Is Essential for the GSK3β-Mediated Degradation of AHR in HeLa Cells

**Figure 7:**
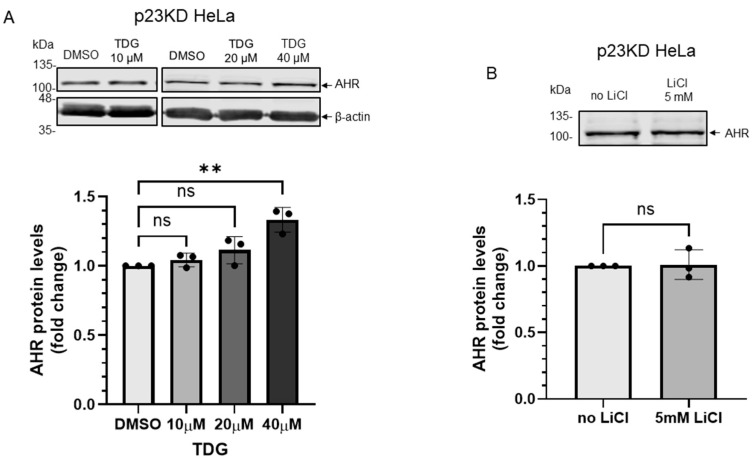

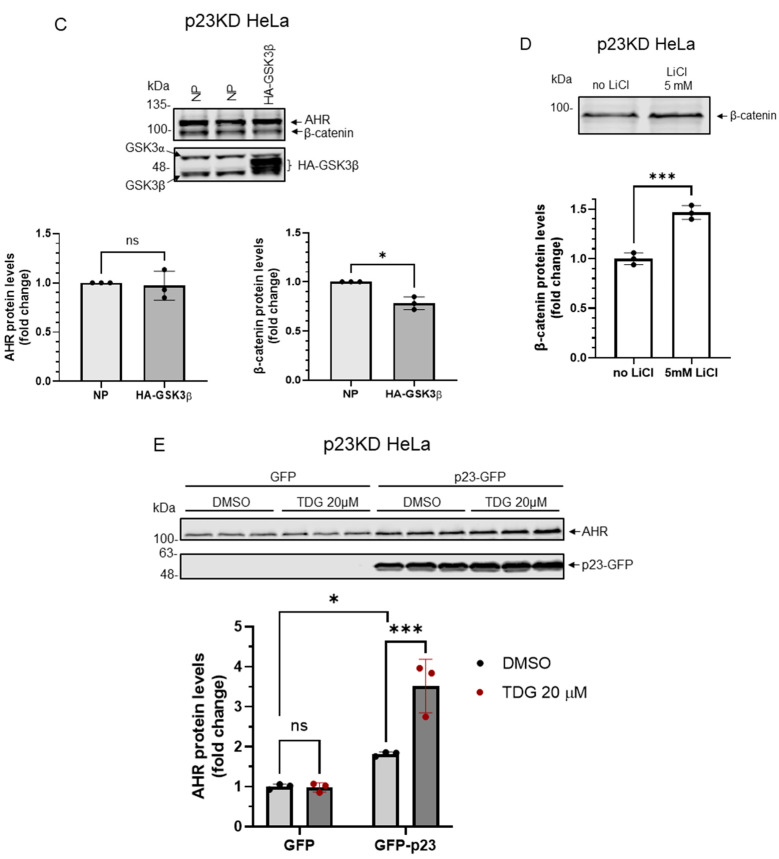
Down-regulation of p23 in HeLa cells causes AHR not responsive to GSK3β regulation. (**A**) AHR protein levels were not altered by 8-h treatment of 10 μM or 20μM TDG in p23 stable knockdown (p23KD) HeLa cells. Treatment of 40 μM TDG increased AHR protein levels to 1.3-fold. The images are representative of the replicate data (means ± SD, *n* = 3, ** *p* < 0.01, ns represents not significant). DMSO group (treated for 8 h) was arbitrarily set as one (with no error bar) for data normalization. One-way ANOVA with Dunnett multiple comparisons test was performed to determine statistical significance. (**B**) Treatment of 5 mM LiCl for 6 h did not alter AHR protein levels in p23KD HeLa cells. The images are representative of the replicate data (means ± SD, *n* = 3, ns represents not significant). No LiCl (no treatment) group was arbitrarily set as one (with no error bar) for data normalization. Unpaired *t*-test with Welch’s correction was used to determine the statistical significance. (**C**) β-catenin protein levels decreased while AHR protein levels showed no significant difference after transient expression of HA-GSK3β (72 h after transient transfection) in p23KD HeLa cells. The images are representative of the replicate data (means ± SD, *n* = 3, *p < 0.05, ns represents not significant). No plasmid group (NP) represents cells undergoing the same transient transfection protocol without the HA-GSK3β expressing plasmid and was arbitrarily set as one (with no error bar) for data normalization. Unpaired *t*-test with Welch’s correction was used to determine the statistical significance. (**D**) β-catenin protein levels increased in p23KD HeLa cells after treatment of 5 mM LiCl for 6 h. The images are representative of the replicate data (means ± SD, *n* = 3 in one experiment, which was repeated once with similar results, *** *p* < 0.001). No LiCl (no treatment) group was arbitrarily set as one for data normalization. Unpaired *t*-test was used to determine the statistical significance. (**E**) p23KD HeLa cells were transiently transfected with the plasmid expressing GFP or GFP-p23. Transient expression of GFP-p23 caused AHR protein levels to increase when cells were treated 20 μM TDG for 8 h. The images are representative of the replicate data (means ± SD, *n* = 3 in one experiment, which was repeated once with similar results, * *p* < 0.05, *** *p* < 0.001, ns represents not significant). DMSO treatment group of GFP-transfected cells was arbitrarily set as one for data normalization. Two-way ANOVA with Sidak multiple comparisons test was performed to determine statistical significance. For A to E, each Western lane contained 30 μg of whole-cell lysate. Data in A were normalized by β-actin (as shown) whereas the rest of the data were normalized by total protein stain.

We previously reported that the basal AHR protein levels are regulated by selective autophagy and this degradation is more pronounced in the stable p23 knockdown (p23KD) HeLa cells, resulting in about 50% of the AHR content when compared to the wild type content [23]. When we treated the p23KD HeLa cells with TDG (10–40 μM), only 40 μM TDG increased AHR protein level significantly to 1.3-fold (Figure 7A), which was lower than what we observed in HeLa cells (1.7-fold, Figure 2A). LiCl (5 mM), on the other hand, could not alter the AHR protein levels in p23KD HeLa cells (Figure 7B). Transient expression of the HA fusion of GSK3β in p23KD HeLa cells decreased the β-catenin protein levels as expected. However, the AHR protein levels were not altered (Figure 7C). Consistently, LiCl caused an increase of the β-catenin protein levels in p23 KD HeLa cells (Figure 7D) but elicited no effect on the AHR protein levels. To further confirm that p23 is essential for the GSK3β effect on AHR, we examined whether this GSK3β effect could be observed in p23 knockdown cells if the p23 levels were restored. We transiently transfected the plasmid expressing the GFP fusion of p23 into p23 stable knockdown HeLa cells and confirmed the expression of p23-GFP (Figure 7E). After transient expression of p23-GFP, the AHR protein levels became sensitive to 20 μM TDG treatment: the AHR protein levels were increased to 1.8-fold, which was similar to the response we observed in wild type HeLa cells (Figure 2), supporting that this AHR regulation by GSK3β is p23-dependent.

### 2.6. GSK3β-Mediated AHR Phosphorylation Is Necessary for Optimal Activation of the AHR Target Gene Transcription in Human Cervical, Liver, and Breast Cancer Cells

**Figure 8:**
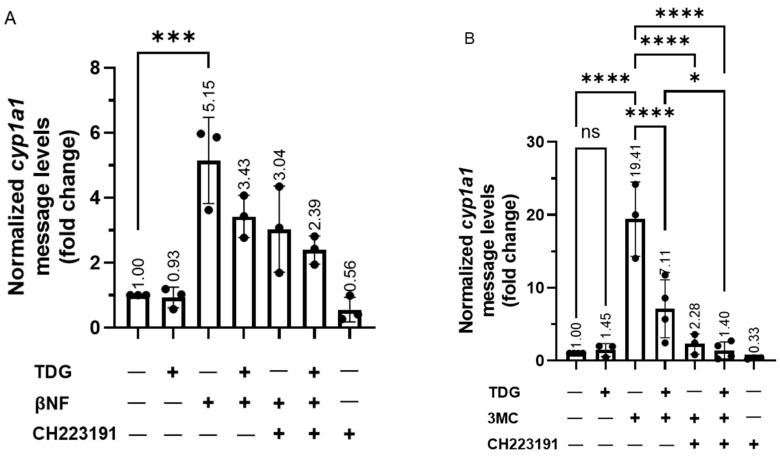

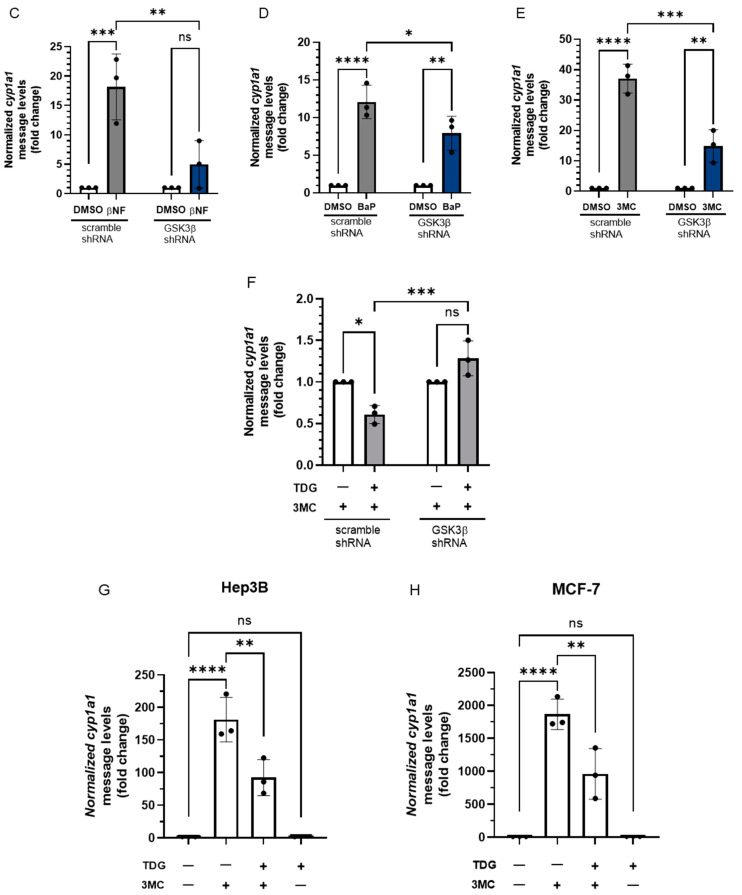
Reduction of GSK3β activity suppresses the ligand-dependent activation of AHR target gene transcription. For A and B, HeLa cells were treated with 40 μM TDG for 9 h. At 5-h post-TDG treatment, cells were treated with either (**A**) 10 μM βNF, 10 μM CH223191 or both or (**B**) 1 μM 3MC, 10 μM CH223191 or both. TDG were able to partially block the increase of the *cyp1a1* message levels triggered by βNF and 3MC. Co-treatment of TDG with CH223191 further decreased the *cyp1a1* message levels. For A and B, the graphs represent replicate data (means ± SD, *n* = 3 except that *n* = 4 for DMSO, TDG + 3MC and TDG + 3MC + CH223191 groups in 8B, * *p* < 0.05, *** *p* < 0.001, **** *p* < 0.0001) and DMSO group was arbitrarily set as one (with no error bar) for data normalization. HeLa cells were transiently transfected for 70 h with the plasmid carrying scramble shRNA or GSK3β specific shRNA. Cells were then treated with (**C**) DMSO or 10 μM βNF, (**D**) DMSO or 5 μM BaP, (**E**) DMSO or 1 μM 3MC for 4 h. Cells were harvested at 74-h post transfection/4-h post ligand treatment. The increase level of the *cyp1a1* message after ligands treatment were significantly lower in GSK3β knockdown groups. For C to E, graphs represent replicate data (means ± SD, *n* = 3, * *p* < 0.05, ** *p* < 0.01, *** *p* < 0.001, **** *p* < 0.0001) with DMSO group arbitrarily set as one (with no error bar) for data normalization. (**F**) 7.5-h treatment of TDG (40 μM, treated at 70-77.5 h post-transfection) could not suppress the 3MC (1 μM, 3.5 h, treated at 74-77.5 h post-transfection) induced *cyp1a1* messages in GSK3β shRNA transfected HeLa cells as observed in scramble shRNA transfected HeLa cells. The graph represents the replicate data (means ± SD, *n* = 3, * *p* < 0.05, *** *p* < 0.001, ns represents not significant) with 3MC groups minus TDG were arbitrarily set as one (with no error bar) for data normalization. Hep3B (**G**) and MCF-7 (**H**) cells were treated 40 μM TDG for 9 h. At 5-h post-TDG treatment, cells were treated with 1 μM 3MC for 4 h. The induced *cyp1a1* message levels were suppressed by TDG in both cell lines. The graphs in G and H represent replicate data (means ± SD, *n* = 3, ** *p* < 0.01, **** *p* < 0.0001, ns represents not significant) with DMSO group arbitrarily set as one (with no error bar) for data normalization. For A to H, one-way ANOVA with Sidak multiple comparisons test was performed to determine statistical significance.

Next, we examined whether the AHR function could be regulated by GSK3β. To do this, we measured the prototypical induction of the cytochrome P450 1A1 gene transcription by an AHR ligand in HeLa cells in the presence or absence of TDG. We observed that the cytochrome P450 1A1 (*cyp1a1*) transcript was induced in HeLa cells, as expected, by two well-known AHR ligands beta-naphthoflavone (βNF) and 3-methylcholanthrene (3MC) to 5- and 19-fold, respectively, and this induction was suppressed effectively by a classical AHR antagonist CH223191 (Figure 8A,B). However, TDG alone did not alter the *cyp1a1* transcript levels but significantly suppressed the βNF (5-fold to 3-fold) and 3MC (19-fold to 7-fold) dependent *cyp1a1* gene transcription. The effect of TDG and CH223191 was synergistic, which was particularly apparent in the case of the 3MC-induced *cyp1a1* gene transcription. This observation suggested that TDG and CH223191 likely inhibit the AHR function via different mechanisms. Downregulation of GSK3β expression via a GSK3β specific shRNA in HeLa cells significantly suppressed the ability of an AHR ligand (βNF, benzo[a]pyrene (BaP) or 3MC) to induce the *cyp1a1* gene transcription (Figure 8C-E). Furthermore, knockdown of GSK3β abolished the TDG suppression of the 3MC-mediated AHR activity (Figure 8F). In addition to what we observed in HeLa cells, this suppression of the 3MC activation of *cyp1a1* gene transcription by TDG was also observed in Hep3B (180-fold to 92-fold) and MCF-7 (1864-fold to 961-fold) cells (Figure 8G,H). Collectively, our results showed that phosphorylation of AHR by GSK3β is essential for optimal AHR activity in terms of the ligand-activated gene transcription.

## 3. Discussion

GSK3β generally regulates its substrate stability (via phosphorylation) by promoting the binding of E3 ligases, such as β-transducin repeat containing proteins (β-TrCP), and eventually targeting its substrates for proteasomal degradation [28]. We previously discovered that without ligand treatment, AHR protein levels are regulated by selective autophagy while inhibition of proteasomal degradation by MG132 further decreases the AHR protein content [23]. This MG132 effect on AHR has also been observed with the GSK3β-mediated AHR degradation which involves the formation of the K63-linked ubiquitination of AHR, revealing the flexibility of degradation mechanisms that can be triggered by GSK3β phosphorylation.

GSK3β is involved in the regulation of mammalian circadian rhythm [32]. In particular, there are at least two PAS proteins, namely period circadian regulator 2 (PER2) and circadian locomotor output cycles kaput (CLOCK), that are GSK3β substrates [32,33]. These proteins are in the same family of AHR, making it intriguing that AHR is also regulated by GSK3β. Another notable GSK3β substrate is β-catenin. There are many aspects of functional dependence between AHR and β-catenin. AHR has been shown as a β-catenin target gene in prostate cancer cells so that increased β-catenin activity leads to increased AHR expression [34]. However, increased AHR function promotes proteasomal degradation of β-catenin since AHR is a E3 ligase which recognizes β-catenin as one of its substrate proteins for degradation [35]. Functionally speaking, AHR and β-catenin operate synergistically to activate the *cyp1a1* gene transcription [36,37]. Interestingly, both AHR and β-catenin activities are controlled by protein degradation mechanisms: AHR undergoes proteasomal degradation after ligand activation whereas β-catenin undergoes proteasomal degradation, limiting its transactivation of the Wnt target genes. Since AHR contains several stretches of serine and threonine residues in its transactivation region which are similar to the GSK3β phosphorylation sites of β-catenin, we explored whether GSK3β would play any role in AHR degradation. Surprisingly, but interestingly, GSK3β causes AHR degradation via autophagy rather than the ubiquitin-proteasome system. In an effort to validate the degradation mechanism, we captured proteasomal degradation using MG132 by proving that the levels of β-catenin were restored upon MG132 treatment. In contrast, MG132 caused reduction of the AHR levels when GSK3β was inhibited by TDG, clearly supporting a mechanism that is different from β-catenin degradation. This reduction is explained by the fact that MG132 can trigger autophagy [38], resulting in further degradation of AHR. One may argue that accumulation of AHR could still be proteasome-mediated since increased p62 levels, which is caused by an autophagy inhibitor such as CQ, has been shown to increase the half-life of proteasome substrates [39]. However, while such substrates accumulate after MG132 treatment [39], AHR did not, supporting that AHR undergoes lysosomal degradation via autophagy. We observed a higher autophagic flux in HA-GSK3β expressing HeLa cells. GSK3β phosphorylates HIV Tat-interactive protein 60 kDa (TIP60), which in turn activates Unc-51 like kinase-1 (ULK1), a key autophagy-related protein that plays an important role in autophagy initiation. Through this GSK3β-TIP60-ULK1 pathway, autophagy is induced under ER-stress or growth factor deprivation [40,41]. However, if the GSK3β-mediated degradation of AHR were merely caused by induction of autophagy, p23 knockdown HeLa cells would have been more pronounced in the suppression of the AHR levels since AHR is more prone to degradation via autophagy when p23 is down-regulated [23]. On the contrary, the GSK3β-mediated AHR degradation is less apparent when the p23 levels are downregulated. The relationship between GSK3β and p23 in affecting AHR degradation is reminiscent of the role of Axin, which is part of the β-catenin destruction complex, in facilitating the GSK3β-mediated degradation of β-catenin [42]. Perhaps p23 plays a role in conforming the structure of AHR to be recognizable by GSK3β. Collectively, our results are consistent with the conclusion that GSK3β phosphorylates AHR in a p23-dependent manner and in turn causes its lysosomal degradation, which is heightened by active GSK3β.

The primary sequence of AHR has multiple locations containing two or more serine and threonine residues that are 3-4 amino acids apart—the minimal requirement for GSK3β recognition. Among them, there are three AHR regions that match the consensus GSK3β targeting sequence the best. We performed mutational analysis to convert every serine or threonine residue into alanine in all three (M1-M3) regions. Results from our deletion studies using CΔ553 (amino acid 1-295) and NΔ515 (amino acid 516-848) revealed that amino acids between 296 and 515 are likely essential for the GSK3β-mediated degradation of AHR since both AHR deletion construct levels were unaltered upon TDG treatment. We, however, cannot rule out the possibility that protein folding could be distorted in these deletion constructs, preventing them to be recognized for lysosomal degradation. Nevertheless, since M1 is located within this 296-515 region, we initially predicted that M1, but not M2 and M3, would contain the GSK3β phosphorylation sites. We also realized that more than one region could be phosphorylated by GSK3β since Shaggy, a drosophila orthologue of GSK3β, phosphorylates Cubitus interruptus (Ci) at two distinct regions that are more than 30 amino acids apart [43]. Interestingly, all three mutants (M1-M3) showed some resistance to GSK3β inhibition by TDG with the order of M2 > M3 ~ M1, suggesting that M2 (rather than M1) likely contains the GSK3β phosphorylation sites. Moreover, all three mutants showed significant, but not complete, resistance to lysosomal degradation by CQ, suggesting that all three regions could mediate AHR lysosomal degradation. GSK3β phosphorylation often works in conjunction with another kinase, since GSK3β substrates are usually “primed” by a kinase, followed by GSK3β phosphorylation [28]. However, the interplay among kinases targeting M1-M3 is much more complicated since the reported “primed” kinase site is only few amino acids away from the GSK3β site, which cannot explain how M1–3 sites could mutually affect one another. We cannot rule out the possibility that M1 and M3 regions could be targeted by a kinase which might somehow direct AHR toward lysosomal degradation, or abrogation of phosphorylation at the M1/M3 mutated regions by a kinase might cause a conformational change that would interfere with the GSK3β phosphorylation at the M2 region. On the contrary, phosphorylation of AHR by GSK3β could cause a conformational change that would allow another kinase to further phosphorylate AHR.

**Figure 9:**
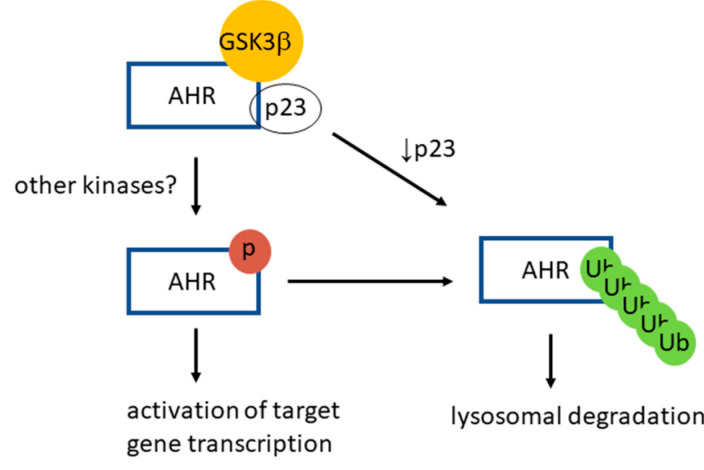
A proposed model of GSK3β role on AHR function and degradation. AHR is phosphorylated by GSK3β in a p23-dependent manner in HeLa cells. This phosphorylation is required for optimal activation of the ligand-dependent AHR target gene transcription. After phosphorylation, AHR is K63-ubiquitinated and is targeted for the LC3-mediated selective autophagy. When the p23 content is compromised in HeLa cells, AHR is more prone to degradation via autophagy, bypassing the GSK3β phosphorylation of AHR.

Inhibition of extracellular signal-regulated (Erk) kinase by inhibitors, such as U0126 and PD98059, increases the AHR protein levels in mouse hepatoma Hepa1c1c7 cells [44]. Expression of a constitutively active form of MEK1 in Hepa1c1c7 cells reduces the AHR protein levels by promoting the Erk kinase-dependent phosphorylation of AHR. Interestingly, these researchers showed that the ligand-dependent function of AHR is suppressed by these Erk kinase inhibitors, similar to what we observed when GSK3β is inhibited or downregulated. However, it is apparent that these Erk inhibitors can physically interfere with the action of a more potent AHR ligand since U0126 is an AHR activator whereas PD98059 is an AHR antagonist [44]. In our case, suppression of the AHR transcriptional activation is likely mediated directly through the action of GSK3β in phosphorylating AHR, since inhibition of GSK3β (by TDG) and downregulation of GSK3β showed similar degrees of suppression. The treatment of TDG in the GSK3β knockdown HeLa cells did not further suppress the AHR transactivation function. Moreover, the putative phosphorylation sites (M1-M3) of AHR overlap with its region required for physical recruitment of coactivators, making it plausible that phosphorylation positively affects the transcriptional activation of AHR [44]. Our results reveal interesting insights on how AHR signals: AHR is more active when it is phosphorylated; in other words, phosphorylation is a means to modulate AHR function. In addition, phosphorylation of AHR is also a trigger for its own lysosomal degradation, i.e., it acts as an “off” switch since the half-life of the phosphorylated AHR is governed by lysosomal degradation (Figure 9). AHR phosphorylation may work in conjunction with the proposed “off” switches of AHR—proteasomal degradation of AHR after ligand activation [21] and upregulation of the AHR repressor *(ahrr)* gene transcription by AHR [45].

## 4. Materials and Methods

### 4.1. Reagents

TDG, CQ, CH223191, 3MC (Sigma, St. Louis, MI, USA), βNF, polybrene, N-ethylmaleimide (NEM), and BaP were purchased from Sigma (St. Louis, MO, USA). LiCl was purchased from Fisher scientific (Rockford, IL, USA). K63-TUBE-biotin, PR-619, and 1,10-phenanthroline were purchased from LifeSensors (Malvern, PA, USA). MG132 was purchased from Cayman Chemical (Ann Arbor, MI, USA). Puromycin was purchased from Gold Biotechnology (St. Louis, MO, USA). GSK3β shRNA, p23 shRNA, and scramble shRNA were purchased from Dharmacon (Lafayette, CO, USA). HA-GSK3β expressing plasmid (1015) was a gift from Scott Friedman (Addgene plasmid # 49491; http://n2t.net/addgene:49491; RRID: Addgene_49491) [46]. pCMV-VSV-G was a gift from Bob Weinberg (Addgene plasmid # 8454; http://n2t.net/addgene:8454; RRID: Addgene_8454). pCMV-dR8.2 dvpr was a gift from Bob Weinberg (Addgene plasmid # 8455; http://n2t.net/addgene:8455; RRID: Addgene_8455). The codon humanized pGFP2-N2 plasmid was purchased from BioSignal Packard (Montreal, QC, Canada). EndoFectin transfection reagent was purchased from GeneCopoeia (Rockville, MD, USA). ZymoPURE II Plasmid Maxiprep kit and Direct-zol RNA Miniprep kit were purchased from Zymo Research (Irvine, CA, USA). MMLV high-performance reverse transcriptase was purchased from Epicentre (Madison, WI, USA). iTaq Universal SYBR Green Supermix was purchased from Bio-Rad (Hercules, CA, USA). QuikChange Lightning site-directed mutagenesis kit (210518) was purchased from Agilent (Santa Clara, CA, USA). Phos-tag acrylamide (AAL-107) was purchased from FUJIFILM Wako Chemicals (Richmond, VA, USA). λ-PP, anti-GSK3α/β mouse IgG (0011-A), anti-β-catenin mouse IgG (15B8), and anti-GFP mouse IgG (B-2) were purchased from Santa Cruz Biotechnology (Dallas, TX, USA). Protein G Dynabeads and anti-p23 antibody (JJ3) were purchased from ThermoFisher Scientific (Rockford, IL, USA). Anti-AHR SA-210 rabbit polyclonal antibody was purchased from Enzo Life Sciences (Farmingdale, NY, USA). Anti-β-actin mouse monoclonal antibody (AM4302) was purchased from Ambion (Austin, TX, USA). Anti-LC3B rabbit IgG (L7543) and IgG from rabbit serum were purchased from Sigma-Aldrich (St. Louis, MO, USA). Nitrocellulose membrane, REVERT 700 Total Protein Stain for Western blot normalization, IRDye 800CW, IRDye 680 anti-rabbit, anti-mouse secondary antibody, and IRDye 800CW streptavidin were purchased from LI-COR Bioscience (Lincoln, NE, USA). GlutaMAX-I, penicillin, and streptomycin were purchased from Invitrogen (Carlsbad, CA, USA). HyClone FBS and HyClone DMEM were purchased from Fisher scientific (Rockford, IL, USA).

### 4.2. Cell Culture

HeLa and Hep3B cell lines were authenticated by ATCC. MCF-7 cell line was obtained from ATCC. AD-293 cells were obtained from Agilent Technologies (Santa Clara, CA, USA). All cell lines were maintained in DMEM supplemented with 10% fetal bovine serum, 2 mM GlutaMAX-I, 10 U/mL of penicillin, and 10 mg/mL of streptomycin at 37 °C and 5% CO_2_.

### 4.3. Generation of p23 Stable Knockdown Cells

We used our previously published protocol [23] to generate new p23 stable knockdown HeLa cells for this study. In brief, on day 1, AD-293 cells (about 7 × 10^5^ cells) were seeded in 5 mL of DMEM (10% FBS) without antibiotics in a 25 cm^2^ flask and incubated at 37 °C and 5% CO_2_ overnight. On day 2, cells should reach 50–80% confluence and in fresh medium without antibiotics. Transfection was performed in the late afternoon using EndoFectin transfection reagent (2:1 DNA ratio) with the plasmid cocktail as follows: 2.5 μg of p23 shRNA plasmid (#1475), 1.875 μg of the pCMV-dR8.2 dvpr packaging plasmid, and 0.625 μg of the VSV-G envelope plasmid. Fresh complete medium was exchanged 15 h after transfection (day 3). After 24 h, medium which contained the virus was collected (day 4) and stored at 4 °C. Another 5 mL of fresh complete medium was added to cells and was collected 24 h afterwards (day 5). The combined medium was centrifuged at 400× *g* for 5 min to remove any AD-293 cells that were inadvertently collected. The resulting supernatant was used for infection. Lentiviral infection of HeLa cells was performed by first seeding cells in a 75 cm^2^ flask to 50–70% confluence. Fresh complete medium containing 8 μg/mL of polybrene was exchanged. Supernatant containing lentiviral particles (0.5 mL) was then added. The fresh complete medium was exchanged 24 h after infection. The selection was started by adding 1.5 μg/mL of puromycin 48–54 h after infection. Western analysis was performed to determine the p23 protein levels after 2–3 passages.

### 4.4. Transient Transfection

Cells were grown in 6-well plates overnight (16-18 h) and transfection was initiated when cells were about 90-95% confluence. Cells were transfected with 3 μg of plasmid (4 μg for HA-GSK3β) and 6 μL of EndoFectin reagent (8 μL for HA-GSK3β). Fresh complete medium was exchanged 24 h after transfection. HA-GSK3β and GSK3β shRNA plasmids were transfected for 72 h. Usually, we initiated cell treatment at 70 h post transfection. pGFP plasmids were transfected for 48 h with cell treatment initiated at 48 h post transfection.

### 4.5. Whole-Cell Lysate Preparation

Cells were harvested using cold 1xPBS by mechanical scrapping. Cell pellets were resuspended using cold lysis buffer (25 mM HEPES, pH 7.4, 0.4 M KCl, 1 mM EDTA, 1 mM DTT, 10% glycerol, 1% NP-40, 1 mM PMSF, and 2 μg/mL of leupeptin) of 3 times the volume of cell pellets. After three cycles of freeze/thaw, lysates were kept on ice for 30 min and were then centrifuged at 16,000× *g* for 10 to 20 min at 4 °C. The supernatants were defined as whole-cell lysates and were subjected to BCA assay to determine the protein concentration.

### 4.6. Western Blot Analysis

The protocol for Western blot analysis was described previously [23] with minor modification. In brief, if 15% instead of 10% acrylamide gels were used, they were transferred via full submersion for an additional one h (3 h total) at 4 °C. After the wet transfer, total protein staining was performed using LI-COR REVERT Total Protein Stain. Membranes for the examination of LC3B levels were dried for at least 40 min and wet with PBS before blocking. The transferred nitrocellulose membranes were blocked in PBS with 5% BSA for 1 h. Dilutions for antibodies were as follows: 1:1000 for anti-p23 JJ3 and anti-LC3B L7543; 1:2000 for anti-AHR SA210; 1:5000 for anti-β-actin AM4302; 1:200 for anti-GSK3β 0011-A and anti-β-catenin 15B8. If not specified, Western bands were normalized using total protein stain. Results were obtained and analyzed using a LI-COR Odyssey CLx imaging system.

### 4.7. RT-qPCR

RT-qPCR was performed as described previously [47]. In brief, when HeLa cells in a 75 cm^2^ flask reached 90–100% confluence, 5% of cells (0.3 mL of 6 mL total cell suspension) were seeded onto each well of a 6-well plate. After incubation for 16–18 h, cells were treated with different reagents or transfected with the GSK3β shRNA at 90–95% confluence. For Hep3B and MCF-7 cells, when cells in 75 cm^2^ flask reached 95% confluence, 8.3% of cells (0.5 mL of 6 mL total cell suspension) were seeded onto each well of a 6-well plate. After incubation for about 24 h, cells reached 80–90% confluence and were treated with different reagents. After treatment, media were aspirated, and RNA was extracted using the Direct-zol kit with TRI reagent. Reverse transcription was performed for 1 μg of RNA using Epicentre MMLV reverse transcriptase. Quantitative PCR was performed with: 1 μL of cDNA from reverse transcription solution, 10 μL of Bio-Rad iTaq SYBR green supermix, and 0.8 pmol sequence-specific primers (*cyp1a1* primers are OL109, 5′-GGC CAC ATC CGG GAC ATC ACA GA-3′ and OL110, 5′-TGG GGA TGG TGA AGG GGA CGA A-3′; *β-actin* primers are OL101, 5′-CCA CAC TGT GCC CAT CTA GG-3′ and OL102, 5′-AGG ATC TTC ATG AGG TAG TCA GTC AG-3′) using a Bio-Rad CFX Connect real-time PCR machine with the following protocol: 40 cycles of 90 °C for 10 s/60 °C for 1 min with fluorescence readings taken at 60 °C. The 2 ^−∆∆Cq^ method [48] was used to present the normalized values.

### 4.8. Immunoprecipitation and K63-TUBE Far-Western Analysis

HeLa cells were seeded and cultured overnight in 6-well plates. Transfection of the GSK3β shRNA was initiated when cells reached about 90% confluence and was continued for 72 h. Immunoprecipitation was performed as described previously [23]. In brief, cells were lysed as described under 4.5 with 4 times the volume of pellet size. Three deubiquitylase inhibitors, namely 1,10-phenanthroline (5 mM), NEM (10 mM), and PR-619 (50 μM) were added in the lysis buffer for immunoprecipitation experiment with K63-TUBE Far-western analysis. About 1.5-2.0 milligrams of whole-cell lysates were used for immunoprecipitation of AHR using the anti-AHR SA210 antibody (1:200 by volume) for 30 min at room temperature. The pre-equilibrated Protein G Dynabeads (1:200 by volume) were then added to each sample with the assay buffer: 25 mM HEPES, pH 7.4, 0.15 M NaCl, 1 mM EDTA, 1 mM DTT, 10% glycerol, 0.1% Tween-20, and 1 mg/mL of BSA. The samples were incubated with a rotation of 60 rpm overnight at 4 °C. The beads were then washed three times for 5 min each with the assay buffer and then eluted with electrophoresis sample buffer for SDS-PAGE, followed by Far-western analysis. K63-TUBE biotin (1:1000) were incubated with the nitrocellulose membrane for 1 h at room temperature and then with IRDye-800 conjugated streptavidin (1:10,000) for 2 h at room temperature. The wash step between incubation was the same as in Western analysis. Results were obtained and analyzed using a LI-COR Odyssey CLx imaging system.

### 4.9. Phos-Tag Gel SDS-PAGE

Whole-cell lysates were treated with 0, 80 units (1 μL), or 400 units (5 μL) of λ-PP for 2, 30, or 60 min at 30 °C. To detect the phosphorylated AHR and β-catenin, a gel mix containing 7.5% acrylamide, 20 μM phos-tag reagent, and 80 μM ZnCl_2_ was prepared to make the phos-tag acrylamide gel, according to the manufacturer’s protocol. Bis-Tris SDS-PAGE system was used with MOPS as the running buffer. SDS-PAGE were performed at 4 °C; 55 volts for the stacking gel and 110 volts for the resolving gel. Wet transfer was performed at 4 °C for 4 h. Western blot analysis was performed as described under 4.6.

### 4.10. Site-Directed Mutagenesis

QuikChange II lightning kit was used to perform site-directed mutagenesis. All the procedures were performed strictly according to the manufacturer’s protocol. For the mutant strand synthesis reaction, 10 ng of the pGFP2-N2-AHR plasmid [24] were used as the template. Primers are listed in Table 1. The full-length cDNA sequences of all mutant plasmids were confirmed by sequencing (Functional Biosciences, Madison, WI).

### 4.11. Statistical Analysis

GraphPad Prism 9 software (La Jolla, CA, USA) was utilized for statistical analysis. Two-tailed unpaired t-test and one-wa ay and two-way (or mixed-model) ANOVA with Sidak, Tukey or Dunnett tests for multiple comparisons were used to determine statistical significance with * *p* < 0.05, ** *p* < 0.01, *** *p* < 0.001, **** *p* < 0.0001, and ns, not significant (*p* > 0.05). Some specific detail is also mentioned in the figure legends.

## 5. Conclusions

Phosphorylation of AHR by GSK3β occurs in HeLa cells even without exogenous ligand treatment. After the GSK3β-mediated phosphorylation, AHR undergoes lysosomal degradation and is more active in the ligand-activated gene transcription. Inhibition of GSK3β activity by TDG suppresses the transcriptional activation function of AHR in at least HeLa, Hep3B, and MCF-7 cells, suggesting that the role of GSK3β in AHR modulation is likely a general mechanism across many human cell types.

## Figures and Tables

**Table 1:** M1, M2, and M3 using the pGFP2-N2-AHR plasmid as the template for QuikChange mutagenesis.

Target Sequence	Primer Sequence
S436A-S440A-S444A	Forward(OL904): 5′-a aat ggc act gct gga aaa gac gct gct acc aca gcc act cta agc aag g -3′
	Reverse(OL905): 5′-c ctt gct tag agt ggc tgt ggt agc gtc ttt tcc agc agt gcc att t-3′
S689A-S693A-T697A	Forward(OL906): 5′-gag ttc ccc tac aaa gct gaa atg gat gct atg cct tat gca cag aac ttt att tcc-3′
	Reverse(OL907): 5′-gga aat aaa gtt ctg tgc ata agg cat agc atc cat ttc agc ttt gta ggg gaa ctc-3′
S723A-S727A-T731A	Forward(OL908): 5′-c tac cct atg ggg gct ttt gaa cca gcc cca tac ccc gct act tct agt t-3′
	Reverse(OL909): 5′-a act aga agt agc ggg gta tgg ggc tgg ttc aaa agc ccc cat agg gta g-3′

## Abbreviations

AHR	aryl hydrocarbon receptor
ARNT	aryl hydrocarbon receptor nuclear translocator
GSK3β	glycogen synthase kinase 3 beta
Erk kinase	extracellular signal-regulated kinase
β-TrCP	β-transducin repeat containing proteins
CQ	chloroquine
TDG	Tideglusib
LiCl	lithium chloride
NEM	N-ethylmaleimide
HA-GSK3β	HA fusion of GSK3β
TUBE	specific tandem ubiquitin binding entity
K63	lysine 63
λ-PP	λ-phosphatase
p23KD	p23 knockdown
3MC	3-methylcholanthrene
βNF	beta-naphthoflavone
BaP	benzo[a]pyrene
NP	no plasmid
cyp1a1	cytochrome P450 1A1
TIP60	HIV Tat-interactive protein 60 kDa
ULK1	Unc-51 like kinase-1
ahrr	AHR repressor
